# Crystal structure of 1,2-bis­(4-fluoro­phen­yl)-1-hy­droxy-2,3,8-tri­meth­oxy­acenaphthene: formation of a five-membered intra­molecular O—H⋯O hydrogen-bonded ring

**DOI:** 10.1107/S2056989021000669

**Published:** 2021-01-26

**Authors:** Hiroaki Iitsuka, Kun Li, Miyuki Kobayashi, Kikuko Iida, Noriyuki Yonezawa, Akiko Okamoto

**Affiliations:** a Department of Organic and Polymer Materials Chemistry, Tokyo University of Agriculture & Technology (TUAT), Koganei, Tokyo 184-8588, Japan

**Keywords:** acenaphthene, asymmetric mol­ecular structure, *cis*-configuration, intra­molecular O—H⋯O hydrogen bond, crystal structure

## Abstract

The structure of the title compound, C_27_H_22_F_2_O_4_, at 193 K has triclinic (*P* ) symmetry. The hy­droxy and meth­oxy groups at the 1,2-positions of the acenaphthene core display a *cis* configuration. Both substituents are involved in the formation of a five-membered intra­molecular O—H⋯O hydrogen-bonded ring. The 4-fluoro­phenyl rings make dihedral angles of 87.02 (7) and 51.86 (8)° with the naphthalene ring system. In the crystal, a pair of non-classical C—H⋯O hydrogen bonds forms centrosymmetric dimeric structures. The dimeric aggregates are linked in the *ac* plane through non-classical C—H⋯F hydrogen bonds and C—H⋯π interactions.

## Abstract 2

In the crystal of the title compound, the formation of an intra­molecular O–H⋯O hydrogen bond between the hy­droxy group and the meth­oxy group at the 1,2-positions of the acenaphthene ring core giving rise to a five-membered cyclic organization is observed. In the mol­ecular packing, a pair of non-classical C—H⋯O hydrogen bonds forms centrosymmetric dimeric structures.

## Chemical context   

The chemistry of congested aromatic-ring-accumulation compounds has attracted continuous inter­est, especially in non-classical non-covalent bonding inter­actions other than classical hydrogen bonds. Steric factors of these compounds influenced by the presence of exocyclic bonds presumably bring about in-plane and/or out-of-plane deviations from the ordinary geometry of aromatic mol­ecules. Consequently, the mol­ecules undergo geometrical changes to release the strain in the mol­ecular skeleton, which, in turn, modulates the π-electron delocalization. These space–structural characteristics result in an alteration of the reactivity and properties of the near-by moiety of the mol­ecule (Tannaci *et al.*, 2007 ▸; Pascal, 2006 ▸; Downing *et al.*, 1994 ▸; Biedermann *et al.*, 2001 ▸). From the point of view of such structural properties, the authors have been investigating *peri*-substituted naphthalene and 1,2-di­substituted acenaphthene compounds, focusing on the mol­ecular structure and packing of the above compounds and their analogues and homologues along with the reaction behaviour, including the formation reaction and the design of novel categories of highly performing and functional organic and polymer materials (Okamoto & Yonezawa, 2015 ▸).

The authors have found that *peri*-aroyl­naphthalene compounds are selectively yielded *via* electrophilic aromatic diaroylation of a naphthalene derivative in the presence of a suitable acidic mediator (Okamoto & Yonezawa, 2009 ▸; Okamoto *et al.*, 2011 ▸). In *peri*-aroyl­naphthalene compounds, probably caused by steric hindrance, the aroyl groups tend to be arranged nearly perpendicular relative to the core naphthalene plane. Bearing this in mind, the authors have continued their crystallographic study of homologous and analogous *peri*-aroyl­naphthalene compounds for elucidation of the correlation between mol­ecular structure, crystal packing and non-covalent bonding inter­actions. As one of the readily performable reactions of *peri*-aroylnaphthalene compounds, a Zn-mediated reductive coupling to 1,2-diaryl-1,2-acenaphthenediol has been discovered (Mido *et al.*, 2017 ▸, 2020 ▸). Herein, the crystal structure of 1,2-bis­(4-fluoro­phen­yl)-1-hy­droxy-2,3,8-tri­meth­oxy­acenaphthene (I), a mono­alk­oxy­l­ated derivative of a pinacol-coupling product, is reported and its structural features are discussed through comparison with homologues.
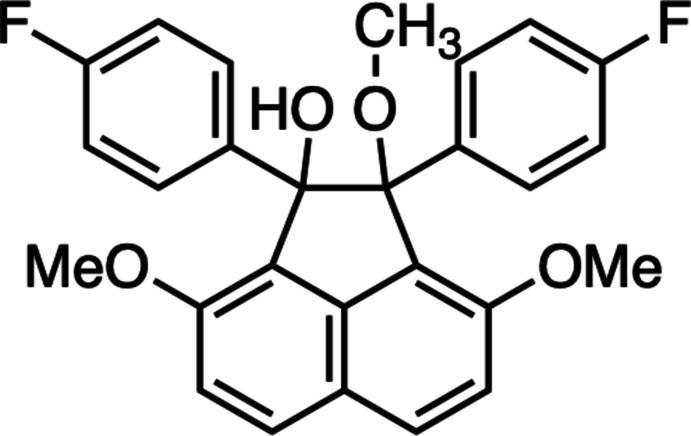


## Structural commentary   

**Figure 1:**
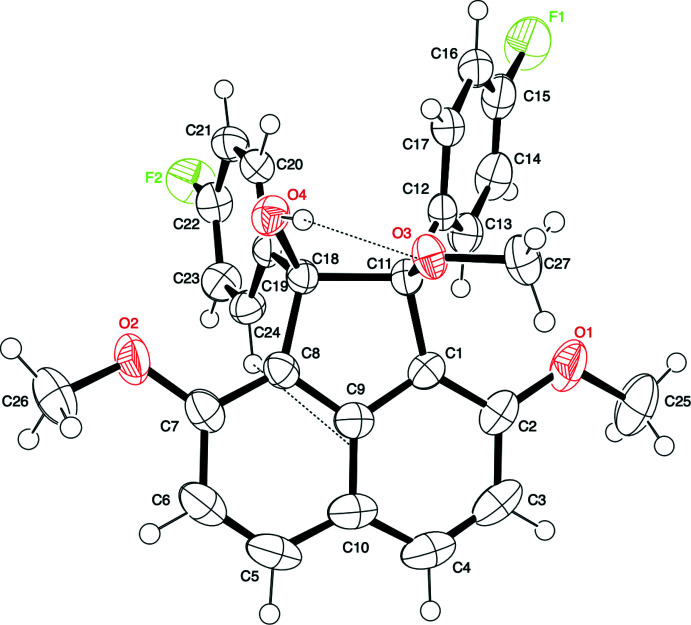
The mol­ecular structure of the title compound, with atom labelling and intra­molecular O—H⋯O and C—H⋯π contacts (dashed lines). Displacement ellipsoids are drawn at the 50% probability level.

**Figure 2:**
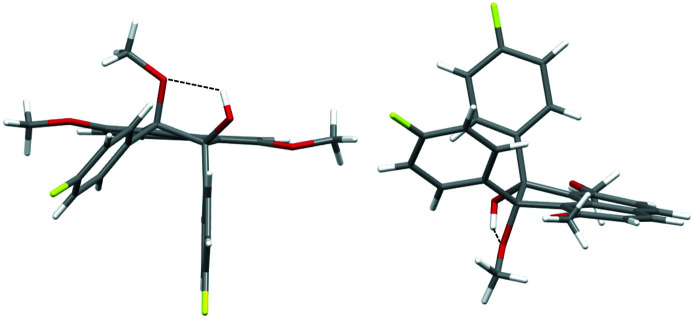
Top (left) and side view (right) of the title compound showing the intra­molecular O—H⋯O contact (dashed lines).

The mol­ecular structure of the title compound is shown in Fig. 1 ▸. This compound consists of an acenaphthene ring system core with a hy­droxy group and a 4-fluoro­phenyl group at the 1-position, a meth­oxy group and a 4-fluoro­phenyl group at the 2-position, and two meth­oxy groups at the 3- and 8-positions. In the title compound, the two 4-fluoro­phenyl groups at the 1,2-positions are located on the same side of the acenaphthene ring system plane (*i.e. cis*), and the 1-hy­droxy and 2-meth­oxy groups are positioned on the other side. Moreover, the hydrogen atom of the hy­droxy group at the 1-position is located in between the two oxygen atoms (O3 and O4) of the hy­droxy and the meth­oxy groups at the 1,2-positions, with the methyl group oriented away. A puckering analysis (Cremer & Pople, 1975 ▸; Luger & Bülow, 1983 ▸) suggests that the five-membered ring of the acenaphthene core has a half-chair conformation. The positions of the ring substituents can be described as bis­ectional for the hy­droxy group at the 1-position, axial for the 4-fluoro­phenyl group at the 1-position, axial for the meth­oxy group at the 2-position, and bis­ectional for the 4-fluoro­phenyl group at the 2-position (Fig. 2 ▸). The two benzene rings of the 4-fluoro­phenyl groups are twisted out of the naphthalene plane (C1–C10) of the acenaphthene ring system. The C19–C24 benzene ring at the 1-position is more heavily tilted compared to the C12–C17 benzene ring at the 2-position, as indicated by the dihedral angles of the best planes through the benzene rings and naphthalene ring system, which are 87.02 (7) and 51.86 (8)°, respectively. The dihedral angle between the two benzene rings is 43.47 (9)°. Furthermore, the C12—C11—C18—C19 torsion angle [31.37 (15)°] indicates a large slippage between the two benzene rings. In addition, the five-membered ring C1,C9,C8,C18,C11 and the naphthalene ring system (C1–C10) are not coplanar, the dihedral angle between their best planes being 7.03 (7)°.

An intra­molecular classical O—H⋯O hydrogen bond forming a five-membered cyclic arrangement is observed between hy­droxy group O4—H4 and oxygen atom O3 of the meth­oxy group at the 1- and the 2-positions of the acenaphthene ring system (see Table 1 ▸). An intra­molecular C—H⋯π inter­action between hydrogen atom H24 of one of the 4-fluoro­phenyl groups and the acenaphthene ring system is also observed (C24—H24⋯*Cg*9 = 2.90 Å; *Cg*9 is the centroid of the acenaphthene ring; see also Table 1 ▸). The possibility of intra­molecular classical O—H⋯O hydrogen bond formation as part of a five-membered cyclic arrangement in 1,2-acenaphthenediol was proposed several decades ago by infrared spectroscopy (Moriconi *et al.*, 1959 ▸; Hayward & Csizmadia, 1963 ▸). Trotter and Mak have designed and synthesized *cis*-1,2-acenaphthenediol (Trotter & Mak, 1963 ▸). However, in the crystal structure of the pinacol compound, no effective intra­molecular inter­actions were observed. Instead, inter­molecular O—H⋯O inter­actions align the mol­ecules sequentially to form a chain-like structure in the crystal packing and the formation of *inter*mol­ecular O—H⋯O hydrogen bonds took precedence over an intra­molecular classical O—H⋯O hydrogen bond. In contrast, in the title compound, the hy­droxy and meth­oxy groups are presumably forced to form an intra­molecular hydrogen bond, *i.e.*, the spatial arrangement of the two benzene rings – probably restricted by the meth­oxy groups at the 3,8-positions – inhibits the approach of other mol­ecules.

## Supra­molecular features   

**Figure 3:**
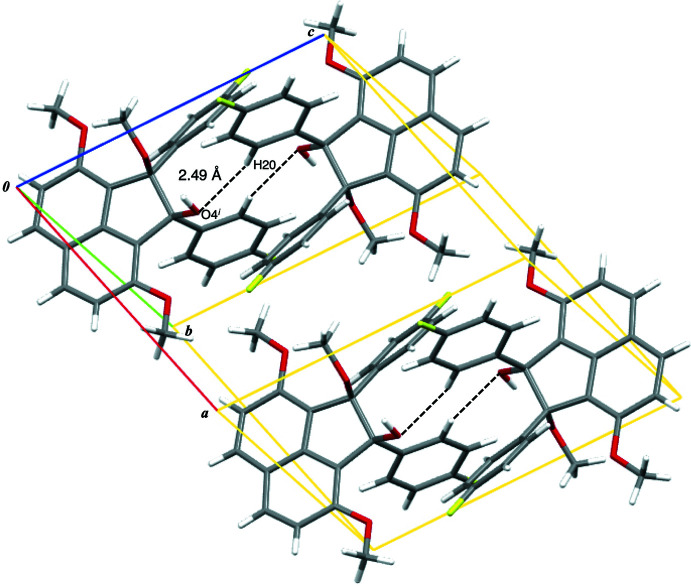
A view of the crystal packing of the title compound, showing the centrosymmetric dimers. The non-classical O—H⋯O hydrogen bonds are shown as dashed lines [symmetry code: (i) −*x*, −*y* + 1, −*z* + 1].

**Figure 4:**
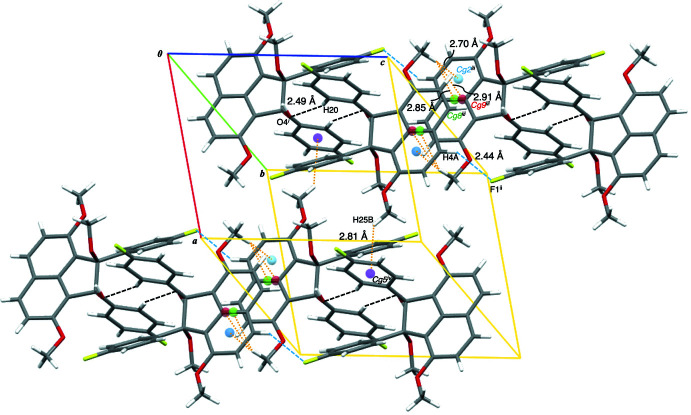
A view of the crystal packing of the title compound, showing the arrangement of the dimeric mol­ecular units. Non-classical C—H⋯F, C—H⋯O and C—H⋯π hydrogen bonds are shown as blue, black and orange dashed lines, respectively [symmetry codes: (i) −*x*, −*y* + 1, −*z* + 1; (ii) *x*, *y*, *z* + 1; (iii) −*x*, −*y* + 1, −*z* + 2; (iv) *x* + 1, *y*, *z*; centroids are defined in Table 1 ▸].

In the mol­ecular packing, a pair of non-classical C—H⋯O hydrogen bonds between hydrogen H20 of a 4-fluoro­phenyl group (2-positioned) and oxygen O4 of the hy­droxy group at the 1-position of the acenaphthene unit connects two mol­ecules of the title compound, forming a centrosymmetric dimer [C20—H20⋯O4^i^, 2.49 Å; symmetry code: (i) −*x*, 1 − *y*, 1 − *z*] (Table 1 ▸, Fig. 3 ▸). The dimers are arranged along the *c* axis through non-classical C—H⋯F hydrogen bonds between hydrogen atom H4*A* at the 5-position of the acenaphthene unit and fluorine atom F1 of the 4-fluoro­phenyl group at the 2-position of the acenaphthene ring system [C4—H4*A*⋯F1, 2.44 Å; symmetry code: (ii) *x*, *y*, 1 + *z*] (Table 1, Fig. 4 ▸ ▸). In addition, three non-classical C—H⋯π hydrogen bonds between the meth­oxy group at the 8-position and the acenaphthene unit [C26—H26*C*⋯*Cg*2^iii^, 2.70 Å; C26—H26*C*⋯*Cg*8^iii^, 2.85Å; C26—H26*C*⋯*Cg*9^iii^, 2.91 Å; symmetry code: (iii) −*x*, −*y* + 1, −*z* + 2; *Cg*2 and *Cg*8 are the centroids of the rings (C1–C4,C9,C10) and (C1–C10), respectively] (Fig. 4 ▸). The dimer chains are linked by non-classical C—H⋯π hydrogen bonds along the *a-*axis direction [C25—H25*B*⋯*Cg*5^iv^, 2.81 Å; symmetry code: (iv) *x* + 1, *y*, *z*; *Cg*5 is the centroid of ring (C19–C24)] (Fig. 4 ▸).

The asymmetric mol­ecular structure, with only one of the hy­droxy groups meth­oxy­lated, disrupts the spatial alignment observed when both hy­droxy groups inter­act with adjacent mol­ecules, forming chain structures (Trotter & Mak, 1963 ▸). Instead of the stabilization energy obtained by forming a chain structure, the title mol­ecules afford the centrosymmetric dimer as the packing motif. The intra­molecular classical O—H⋯OMe hydrogen bond is required to adjust the spatial arrangement for forming centrosymmetric dimers. The non-classical hydrogen-bonding inter­actions connecting the dimeric aggregates contribute to further stabilize the mol­ecular packing.

## Database survey   

A search of the Cambridge Structural Database (CSD version 5.41, last update August 2020; Groom *et al.*, 2016 ▸) for the 1,2-disubstituted acenaphthene moiety of the title compound yielded 27 hits. These include compounds with a 1,2-acenaphthenediol moiety and a similar 1,2-diaryl-1,2-acenaphthenediol unit. A search for 1,2-acenaphthenediol and its derivatives gave 18 hits (CSD refcode FILQAV: Tao *et al.*, 2018 ▸; FILQEZ: Tao *et al.*, 2018 ▸; GACWUE: Maghsoodlou *et al.* 2009 ▸; QIBMIX: Parvez *et al.* 2001 ▸; HERPIG and HERPOM: Sato *et al.*, 2017 ▸; REWGEG: Jimenez *et al.*, 2007 ▸; ROCBIU: Plater *et al.*, 1997 ▸; TESDIE: Nair *et al.*, 2000 ▸; UYENET, UYENIX and UYENIX01: Joussot *et al.*, 2016 ▸; UYENET01: Joussot *et al.*, 2017 ▸; YIMRIY: Myhill *et al.*, 2018 ▸).

The title compound has a *cis*-configuration. For 1,2-acenaphthenediol and its derivatives, *cis*- and *trans*-configurations are found for 1,2-acenaphthenediol and its dinitrates (ACNAOL: Trotter *et al.*, 1963 ▸; ZZZPKU and ZZZIWC: Mak *et al.*, 1963 ▸; ANADON: Mak *et al.*, 1964 ▸).

A search with a 1,2-diaryl-1,2-acenaphthenediol framework gave nine hits. Among these, three reports are for 1,2-diphenyl-1,2-acenaphthenediol and its clathrates (MOKZER, MOKZIV and MOKZOB: Guo *et al.*, 2000 ▸). In addition we found 1,2-bis­(1′-naphth­yl)-1,2-acenaphthenediol (MOKZUH: Guo *et al.*, 2000 ▸), 1,2-bis­(4-tol­yl)-1,2-acenaphthenediol (CIZTIO: Gatilov *et al.*, 1984 ▸) and 1,2-bis­(4-meth­oxy­phen­yl)-1,2-acenaphthenediol (QARGEW and QATQAE: Suzuki *et al.*, 2005 ▸). Most 1,2-diaryl-1,2-acenaphthenediol derivatives have a *trans*-configuration, except for one example (MOKZUH). In contrast to the title mol­ecule, these 1,2-diaryl-1,2-acenaphthenediol derivatives have a highly symmetric spatial structure, *e.g*., the two phenyl groups make dihedral angles with the naphthalene ring system of 85.42 and 82.93° for CIZTIO, 57.05 and 56.83° for QARGEW, 64.18 and 66.06° for QATQAE *vs* 87.02 (7) and 51.86 (8)° for the title compound. The phenyl rings at the 1,2-positions in these analogues are tilted 20 to 30° from each other, *i.e.*, 22.49° for CIZTIO, 25.88° for QARGEW and 28.10° for QATQAE *vs* 43.47 (9)° for the title compound.

There are only two reports on 1,2-diaryl-1,2-acenaphthene derivatives with both hy­droxy groups protected, *i.e.*, (*S*,*S*,*S*,*S*)-1,2-bis­(4-meth­oxy­phen­yl)acenaphthene-1,2-diyl bis­(2-iso­prop­yl-5-methyl­cyclo­hex­yl) bis­(carbonate) (QARGIA: Suz­uki *et al.*, 2005 ▸) and 1,2-(benz­yloxy)-1,2-bis­(4-chloro­phen­yl)-3,8-di­meth­oxy­acenaphthene (AZOPEL: Takada *et al.*, 2011 ▸). The carbonate analogue (QARGIA) is more similar to 1,2-diaryl-1,2-acenaphthene*diol* than the title compound, with the dihedral angles between the 4-meth­oxy­phenyl rings and the naphthalene ring system being 60.97 and 54.14° and a dihedral angle between the 4-meth­oxy­phenyl rings of 27.17°. The benzyl-protected analogue (AZOPEL) has two similarities with the title compound. First, the spatial arrangement of the 4-phenyl rings with respect to the naphthalene ring system with similar dihedral angles between the 4-chloro­phenyl rings and the naphthalene unit [85.74 (6) and 57.12 (6)° for AZOPEL, 87.02 (7) and 51.86 (8)° for the title compound]. In addition, the formation of centrosymmetric dimers connected by non-classical C—H⋯π hydrogen bonds is observed in the crystal packing.

No reports were found for 1,2-diaryl-1,2-acenaphthene homologues with one hy­droxy group protected.

## Synthesis and crystallization   

1,2-Bis(4-fluoro­phen­yl)-1-hy­droxy-2,3,8-tri­meth­oxy­acenaph­thene (0.25 mmol, 108.6 mg), K_2_CO_3_ (0.25 mmol, 34.55 mg), iodo­methane (0.25 mmol, 35.49 mg) and DMF (0.5 mL) were placed in a 10 mL flask. The reaction mixture was stirred at room temperature for 10 h and then poured into iced water (20 mL). The solution was extracted with CHCl_3_ and successively washed with 2 *M* aqueous HCl and brine. The organic layers thus obtained were dried over anhydrous MgSO_4_. After removal of solvent under reduced pressure, the crude product was obtained (quant.). The cake was crystallized from methanol to give single crystals (isolated yield 64%), m.p. 432–434 K.

^1^H NMR (CDCl_3_, 300 MHz) δ 3.22 (*s*, 3H), 3.73 (*s*, 3H), 3.79 (*s*, 3H), 5.05 (*s*, 1H), 6.48 (*dd*, 4H, *J* = 8.40, 8.40 Hz), 6.73 (*broad*, 4H), 7.21 (*d*, 1H, *J* = 8.70 Hz), 7.24 (*d*, 1H, *J* = 9.00 Hz), 7.82 (*d*, 1H, *J* = 9.00 Hz), 7.88 (*d*, 1H, *J* = 9.00 Hz) ppm.

^13^C NMR (CDCl_3_, 75 MHz) δ 161.92 (*J*_C–F_ = 243 Hz), 161.54 (*J*_C–F_ = 243 Hz), 155.03, 154.46, 141.32, 138.85 (*J*_C–F_ = 2.90 Hz), 133.05 (*J*_C–F_ = 2.90 Hz), 128.51, 128.21 (*J*_C–F_ = 7.88 Hz), 127.20, 127.14 (*J*_C–F_ = 9.30 Hz), 121.93, 119.67, 114.75, 113.79 (*J*_C–F_ = 20.7 Hz), 113.67 (*J*_C–F_ = 22.2 Hz), 113.23, 93.365, 90.248, 56.537, 55.677, 53.440 ppm.

IR (KBr) ν 3488, 2838, 1627, 1601, 1506, 1269, 1224, 1159, 1073, 1048 cm^−1^.

## Refinement   

Crystal data, data collection and structure refinement details are summarized in Table 2 ▸. All H atoms were located in a difference-Fourier map and were subsequently refined as riding on their carriers, with C—H = 0.95 Å (aromatic) and *U*_iso_(H) = 1.2 *U*_eq_(C). Hydrogen atom O4 was refined freely.

## Supplementary Material

Crystal structure: contains datablock(s) I. DOI: 10.1107/S2056989021000669/vm2244sup1.cif

Structure factors: contains datablock(s) I. DOI: 10.1107/S2056989021000669/vm2244Isup2.hkl

IR spectrum of title compound. DOI: 10.1107/S2056989021000669/vm2244sup3.pdf

NMR spectrum (-0.5-10.5 ppm). DOI: 10.1107/S2056989021000669/vm2244sup4.pdf

NMR spectrum (3.0-4.0 ppm). DOI: 10.1107/S2056989021000669/vm2244sup5.pdf

NMR spectrum (6.0-8.0 ppm). DOI: 10.1107/S2056989021000669/vm2244sup6.pdf

Supporting information file. DOI: 10.1107/S2056989021000669/vm2244Isup7.cml

CCDC reference: 2057372

Additional supporting information:  crystallographic information; 3D view; checkCIF report

## Figures and Tables

**Table 1:** Hydrogen-bond geometry (Å, °) *Cg*2, *Cg*5, *Cg*8 and *Cg*9 are the centroids of the rings (C1–C4,C9,C10), (C19–C24), (C1–C10) and (C1–C11,C18), respectively.

*D*—H⋯*A*	*D*—H	H⋯*A*	*D*⋯*A*	*D*—H⋯*A*
O4—H4⋯O3	0.83 (3)	2.00 (3)	2.5119 (18)	119 (3)
C24—H24⋯*Cg*9	0.95	2.90	3.4960 (17)	122
C20—H20⋯O4^i^	0.95	2.49	3.3554 (17)	151
C4—H4*A*⋯F1^ii^	0.95	2.44	3.189 (3)	135
C26—H26*C*⋯*Cg*2^iii^	0.95	2.70	3.614 (2)	155
C26—H26*C*⋯*Cg*8^iii^	0.95	2.85	3.747 (2)	153
C26—H26*C*⋯*Cg*9^iii^	0.95	2.91	3.848 (2)	160
C25—H25*B*⋯*Cg*5^iv^	0.95	2.81	3.527 (3)	131

Symmetry codes: (i) 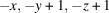; (ii) 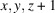; (iii) 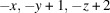; (iv) 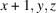.

**Table 2:** Experimental details

Crystal data	
Chemical formula	C_27_H_22_F_2_O_4_
*M*_r_	448.44
Crystal system, space group	Triclinic, *P* 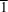
Temperature (K)	193
*a*, *b*, *c* (Å)	10.14886 (18), 11.1827 (2), 11.6411 (2)
α, β, γ (°)	66.724 (1), 77.693 (1), 63.613 (1)
*V* (Å^3^)	1086.07 (4)
*Z*	2
Radiation type	Cu *K*α
μ (mm^−1^)	0.86
Crystal size (mm)	0.8 × 0.35 × 0.1

Data collection	
Diffractometer	Rigaku R-AXIS RAPID
Absorption correction	Numerical (*NUMABS*; Higashi, 1999 ▸)
*T*_min_, *T*_max_	0.542, 0.918
No. of measured, independent and observed [*I* > 2σ(*I*)] reflections	40472, 3919, 3743
*R*_int_	0.063
(sin θ/λ)_max_ (Å^−1^)	0.602

Refinement	
*R*[*F* ^2^ > 2σ(*F* ^2^)], *wR*(*F* ^2^), *S*	0.043, 0.115, 1.06
No. of reflections	3919
No. of parameters	306
H-atom treatment	H atoms treated by a mixture of independent and constrained refinement
Δρ_max_, Δρ_min_ (e Å^−3^)	0.32, −0.21

Computer programs: *PROCESS-AUTO* (Rigaku, 1998 ▸), *CrystalStructure* (Rigaku, 2010 ▸), *SIR2014* (Burla *et al.*, 2007 ▸), *SHELXL2018/3* (Sheldrick, 2015 ▸) and *OLEX2* (Dolomanov *et al.*, 2009 ▸).

## supplementary crystallographic information

### Crystal data

**Table:**

C_27_H_22_F_2_O_4_	*Z* = 2
*M**_r_* = 448.44	*F*(000) = 468
Triclinic, *P*1	*D*_x_ = 1.371 Mg m^−^^3^
*a* = 10.14886 (18) Å	Cu *K*α radiation, λ = 1.54187 Å
*b* = 11.1827 (2) Å	Cell parameters from 15130 reflections
*c* = 11.6411 (2) Å	θ = 4.1–68.2°
α = 66.724 (1)°	µ = 0.86 mm^−^^1^
β = 77.693 (1)°	*T* = 193 K
γ = 63.613 (1)°	Plate, colourless
*V* = 1086.07 (4) Å^3^	0.8 × 0.35 × 0.1 mm

### Data collection

**Table:**

Rigaku R-AXIS RAPID diffractometer	3743 reflections with *I* > 2σ(*I*)
Detector resolution: 10.00 pixels mm^-1^	*R*_int_ = 0.063
ω scans	θ_max_ = 68.2°, θ_min_ = 4.1°
Absorption correction: numerical (NUMABS; Higashi, 1999)	*h* = −11→12
*T*_min_ = 0.542, *T*_max_ = 0.918	*k* = −13→13
40472 measured reflections	*l* = −13→14
3919 independent reflections	

### Refinement

**Table:**

Refinement on *F*^2^	Hydrogen site location: mixed
Least-squares matrix: full	H atoms treated by a mixture of independent and constrained refinement
*R*[*F*^2^ > 2σ(*F*^2^)] = 0.043	*w* = 1/[σ^2^(*F*_o_^2^) + (0.0586*P*)^2^ + 0.398*P*] where *P* = (*F*_o_^2^ + 2*F*_c_^2^)/3
*wR*(*F*^2^) = 0.115	(Δ/σ)_max_ < 0.001
*S* = 1.06	Δρ_max_ = 0.32 e Å^−^^3^
3919 reflections	Δρ_min_ = −0.21 e Å^−^^3^
306 parameters	Extinction correction: SHELXL2018/3 (Sheldrick 2015), Fc^*^=kFc[1+0.001xFc^2^λ^3^/sin(2θ)]^-1/4^
0 restraints	Extinction coefficient: 0.0290 (13)
Primary atom site location: structure-invariant direct methods	

### Special details

**Table:**

Geometry. All esds (except the esd in the dihedral angle between two l.s. planes) are estimated using the full covariance matrix. The cell esds are taken into account individually in the estimation of esds in distances, angles and torsion angles; correlations between esds in cell parameters are only used when they are defined by crystal symmetry. An approximate (isotropic) treatment of cell esds is used for estimating esds involving l.s. planes.

### Fractional atomic coordinates and isotropic or equivalent isotropic displacement parameters (Å^2^)

**Table:**

	*x*	*y*	*z*	*U*_iso_*/*U*_eq_	
F1	0.54395 (13)	0.19439 (16)	0.30239 (12)	0.0761 (4)	
F2	0.10156 (14)	−0.08720 (11)	0.63826 (10)	0.0606 (3)	
O1	0.58174 (13)	0.24840 (15)	0.78038 (13)	0.0578 (4)	
O2	−0.16964 (13)	0.39006 (15)	0.92690 (12)	0.0557 (3)	
O3	0.21992 (11)	0.51843 (10)	0.68218 (9)	0.0355 (3)	
O4	−0.00974 (11)	0.48709 (11)	0.68032 (9)	0.0355 (3)	
C1	0.33402 (16)	0.28835 (15)	0.83979 (13)	0.0348 (3)	
C2	0.47642 (18)	0.23756 (17)	0.87338 (17)	0.0443 (4)	
C3	0.5048 (2)	0.18063 (19)	1.00327 (19)	0.0563 (5)	
H3	0.603535	0.140640	1.026987	0.068*	
C4	0.3953 (2)	0.18154 (19)	1.09462 (17)	0.0547 (5)	
H4A	0.418739	0.145106	1.179868	0.066*	
C5	0.1206 (2)	0.25049 (19)	1.14585 (15)	0.0533 (5)	
H5	0.130872	0.221041	1.233168	0.064*	
C6	−0.0160 (2)	0.30589 (19)	1.10192 (15)	0.0504 (4)	
H6	−0.099321	0.317931	1.158771	0.060*	
C7	−0.03631 (18)	0.34594 (17)	0.97319 (14)	0.0410 (4)	
C8	0.08404 (16)	0.33506 (14)	0.89067 (13)	0.0325 (3)	
C9	0.22314 (17)	0.28432 (15)	0.93596 (13)	0.0352 (3)	
C10	0.2480 (2)	0.23577 (16)	1.06408 (14)	0.0451 (4)	
C11	0.25998 (15)	0.36839 (14)	0.71479 (13)	0.0298 (3)	
C12	0.33911 (15)	0.32035 (15)	0.60487 (13)	0.0315 (3)	
C13	0.44715 (17)	0.18364 (16)	0.62201 (15)	0.0389 (3)	
H13	0.473777	0.119168	0.704339	0.047*	
C14	0.51608 (18)	0.14074 (19)	0.52049 (18)	0.0483 (4)	
H14	0.591121	0.048181	0.532017	0.058*	
C15	0.47354 (18)	0.2349 (2)	0.40290 (17)	0.0498 (4)	
C16	0.36465 (19)	0.3690 (2)	0.38148 (15)	0.0475 (4)	
H16	0.335172	0.430894	0.299057	0.057*	
C17	0.29898 (16)	0.41139 (17)	0.48419 (14)	0.0382 (3)	
H17	0.225034	0.504644	0.471534	0.046*	
C18	0.09992 (14)	0.35988 (14)	0.75182 (12)	0.0293 (3)	
C19	0.09615 (14)	0.23884 (14)	0.72425 (12)	0.0289 (3)	
C20	0.06485 (16)	0.26136 (15)	0.60484 (13)	0.0343 (3)	
H20	0.042476	0.352859	0.542456	0.041*	
C21	0.06587 (18)	0.15193 (17)	0.57558 (14)	0.0403 (4)	
H21	0.044205	0.167574	0.494082	0.048*	
C22	0.09894 (18)	0.02068 (16)	0.66732 (15)	0.0411 (4)	
C23	0.12865 (18)	−0.00576 (16)	0.78620 (15)	0.0428 (4)	
H23	0.149917	−0.097429	0.848094	0.051*	
C24	0.12699 (16)	0.10486 (15)	0.81439 (13)	0.0356 (3)	
H24	0.147274	0.088352	0.896580	0.043*	
C25	0.7328 (2)	0.1777 (3)	0.8115 (3)	0.0758 (7)	
H25A	0.751122	0.222033	0.860643	0.091*	
H25B	0.795464	0.185182	0.734481	0.091*	
H25C	0.755334	0.077256	0.860669	0.091*	
C26	−0.2991 (2)	0.4337 (2)	1.00169 (19)	0.0586 (5)	
H26A	−0.304348	0.350782	1.072245	0.070*	
H26B	−0.385404	0.478465	0.951198	0.070*	
H26C	−0.297245	0.502043	1.033444	0.070*	
C27	0.33921 (18)	0.56172 (17)	0.65522 (16)	0.0439 (4)	
H27A	0.393886	0.521162	0.730991	0.053*	
H27B	0.300590	0.665657	0.626704	0.053*	
H27C	0.405055	0.528052	0.589461	0.053*	
H4	0.010 (3)	0.553 (3)	0.677 (2)	0.068 (7)*	

### Atomic displacement parameters (Å^2^)

**Table:**

	*U*^11^	*U*^22^	*U*^33^	*U*^12^	*U*^13^	*U*^23^
F1	0.0604 (7)	0.1262 (11)	0.0743 (8)	−0.0387 (7)	0.0184 (6)	−0.0758 (8)
F2	0.0874 (8)	0.0489 (6)	0.0625 (6)	−0.0347 (6)	−0.0005 (6)	−0.0285 (5)
O1	0.0283 (6)	0.0732 (8)	0.0771 (9)	−0.0168 (6)	−0.0053 (6)	−0.0335 (7)
O2	0.0382 (7)	0.0851 (9)	0.0588 (7)	−0.0323 (6)	0.0186 (5)	−0.0413 (7)
O3	0.0335 (5)	0.0294 (5)	0.0434 (6)	−0.0157 (4)	0.0060 (4)	−0.0127 (4)
O4	0.0296 (5)	0.0296 (5)	0.0409 (6)	−0.0073 (4)	−0.0062 (4)	−0.0086 (4)
C1	0.0348 (8)	0.0331 (7)	0.0382 (8)	−0.0130 (6)	−0.0062 (6)	−0.0124 (6)
C2	0.0350 (8)	0.0431 (8)	0.0583 (10)	−0.0117 (7)	−0.0108 (7)	−0.0209 (7)
C3	0.0528 (11)	0.0467 (9)	0.0707 (12)	−0.0089 (8)	−0.0329 (9)	−0.0188 (8)
C4	0.0758 (13)	0.0452 (9)	0.0445 (9)	−0.0216 (9)	−0.0256 (9)	−0.0084 (7)
C5	0.0902 (14)	0.0503 (10)	0.0277 (7)	−0.0393 (10)	0.0033 (8)	−0.0118 (7)
C6	0.0710 (12)	0.0550 (10)	0.0382 (8)	−0.0397 (9)	0.0172 (8)	−0.0222 (7)
C7	0.0479 (9)	0.0438 (8)	0.0409 (8)	−0.0272 (7)	0.0111 (7)	−0.0203 (7)
C8	0.0371 (8)	0.0320 (7)	0.0318 (7)	−0.0181 (6)	0.0022 (6)	−0.0113 (5)
C9	0.0440 (8)	0.0304 (7)	0.0334 (7)	−0.0175 (6)	−0.0037 (6)	−0.0094 (6)
C10	0.0682 (11)	0.0370 (8)	0.0346 (8)	−0.0246 (8)	−0.0123 (7)	−0.0075 (6)
C11	0.0269 (7)	0.0278 (6)	0.0334 (7)	−0.0116 (5)	−0.0005 (5)	−0.0090 (5)
C12	0.0256 (7)	0.0362 (7)	0.0364 (7)	−0.0160 (6)	0.0027 (5)	−0.0140 (6)
C13	0.0345 (8)	0.0370 (8)	0.0478 (8)	−0.0144 (6)	−0.0003 (6)	−0.0178 (6)
C14	0.0366 (8)	0.0517 (9)	0.0687 (11)	−0.0154 (7)	0.0028 (8)	−0.0377 (9)
C15	0.0387 (9)	0.0788 (12)	0.0558 (10)	−0.0300 (9)	0.0116 (7)	−0.0461 (9)
C16	0.0409 (9)	0.0744 (12)	0.0370 (8)	−0.0315 (8)	0.0033 (6)	−0.0216 (8)
C17	0.0289 (7)	0.0464 (8)	0.0377 (8)	−0.0158 (6)	0.0018 (6)	−0.0136 (6)
C18	0.0257 (7)	0.0291 (6)	0.0294 (7)	−0.0103 (5)	−0.0008 (5)	−0.0074 (5)
C19	0.0220 (6)	0.0314 (7)	0.0315 (7)	−0.0108 (5)	0.0004 (5)	−0.0096 (5)
C20	0.0345 (7)	0.0357 (7)	0.0315 (7)	−0.0161 (6)	−0.0003 (5)	−0.0086 (6)
C21	0.0445 (9)	0.0480 (9)	0.0342 (7)	−0.0222 (7)	0.0007 (6)	−0.0169 (6)
C22	0.0430 (9)	0.0382 (8)	0.0495 (9)	−0.0191 (7)	0.0036 (7)	−0.0222 (7)
C23	0.0472 (9)	0.0303 (7)	0.0461 (9)	−0.0146 (6)	−0.0064 (7)	−0.0075 (6)
C24	0.0361 (8)	0.0343 (7)	0.0346 (7)	−0.0137 (6)	−0.0059 (6)	−0.0084 (6)
C25	0.0308 (10)	0.0853 (15)	0.1151 (19)	−0.0114 (9)	−0.0110 (10)	−0.0478 (14)
C26	0.0476 (10)	0.0519 (10)	0.0647 (11)	−0.0186 (8)	0.0220 (9)	−0.0228 (9)
C27	0.0444 (9)	0.0433 (8)	0.0531 (9)	−0.0272 (7)	0.0110 (7)	−0.0207 (7)

### Geometric parameters (Å, º)

**Table:**

F1—C15	1.3637 (18)	C12—C17	1.386 (2)
F2—C22	1.3640 (17)	C13—H13	0.9500
O1—C2	1.359 (2)	C13—C14	1.383 (2)
O1—C25	1.428 (2)	C14—H14	0.9500
O2—C7	1.368 (2)	C14—C15	1.370 (3)
O2—C26	1.414 (2)	C15—C16	1.372 (3)
O3—C11	1.4425 (16)	C16—H16	0.9500
O3—C27	1.4273 (18)	C16—C17	1.387 (2)
O4—C18	1.4166 (16)	C17—H17	0.9500
O4—H4	0.84 (2)	C18—C19	1.5269 (18)
C1—C2	1.378 (2)	C19—C20	1.3917 (19)
C1—C9	1.411 (2)	C19—C24	1.3872 (19)
C1—C11	1.5239 (19)	C20—H20	0.9500
C2—C3	1.427 (3)	C20—C21	1.389 (2)
C3—H3	0.9500	C21—H21	0.9500
C3—C4	1.364 (3)	C21—C22	1.372 (2)
C4—H4A	0.9500	C22—C23	1.367 (2)
C4—C10	1.407 (3)	C23—H23	0.9500
C5—H5	0.9500	C23—C24	1.392 (2)
C5—C6	1.362 (3)	C24—H24	0.9500
C5—C10	1.419 (3)	C25—H25A	0.9800
C6—H6	0.9500	C25—H25B	0.9800
C6—C7	1.417 (2)	C25—H25C	0.9800
C7—C8	1.375 (2)	C26—H26A	0.9800
C8—C9	1.401 (2)	C26—H26B	0.9800
C8—C18	1.5133 (18)	C26—H26C	0.9800
C9—C10	1.408 (2)	C27—H27A	0.9800
C11—C12	1.5141 (19)	C27—H27B	0.9800
C11—C18	1.6248 (18)	C27—H27C	0.9800
C12—C13	1.393 (2)		
			
C2—O1—C25	119.03 (16)	F1—C15—C16	118.41 (17)
C7—O2—C26	118.84 (14)	C14—C15—C16	122.98 (15)
C27—O3—C11	116.01 (11)	C15—C16—H16	121.1
C18—O4—H4	106.2 (16)	C15—C16—C17	117.80 (16)
C2—C1—C9	118.08 (14)	C17—C16—H16	121.1
C2—C1—C11	133.46 (14)	C12—C17—C16	121.32 (15)
C9—C1—C11	108.01 (12)	C12—C17—H17	119.3
O1—C2—C1	117.94 (15)	C16—C17—H17	119.3
O1—C2—C3	123.50 (15)	O4—C18—C8	114.24 (11)
C1—C2—C3	118.53 (16)	O4—C18—C11	110.16 (10)
C2—C3—H3	118.8	O4—C18—C19	106.73 (11)
C4—C3—C2	122.39 (16)	C8—C18—C11	101.96 (10)
C4—C3—H3	118.8	C8—C18—C19	112.03 (11)
C3—C4—H4A	119.6	C19—C18—C11	111.78 (10)
C3—C4—C10	120.78 (15)	C20—C19—C18	119.58 (12)
C10—C4—H4A	119.6	C24—C19—C18	121.77 (12)
C6—C5—H5	119.3	C24—C19—C20	118.63 (13)
C6—C5—C10	121.46 (15)	C19—C20—H20	119.5
C10—C5—H5	119.3	C21—C20—C19	120.95 (13)
C5—C6—H6	119.3	C21—C20—H20	119.5
C5—C6—C7	121.39 (16)	C20—C21—H21	120.8
C7—C6—H6	119.3	C22—C21—C20	118.35 (14)
O2—C7—C6	123.20 (14)	C22—C21—H21	120.8
O2—C7—C8	117.86 (14)	F2—C22—C21	118.38 (14)
C8—C7—C6	118.89 (16)	F2—C22—C23	118.95 (14)
C7—C8—C9	119.25 (13)	C23—C22—C21	122.66 (14)
C7—C8—C18	131.42 (14)	C22—C23—H23	120.8
C9—C8—C18	109.19 (12)	C22—C23—C24	118.42 (14)
C8—C9—C1	112.86 (12)	C24—C23—H23	120.8
C8—C9—C10	123.07 (15)	C19—C24—C23	120.98 (14)
C10—C9—C1	124.07 (15)	C19—C24—H24	119.5
C4—C10—C5	128.23 (15)	C23—C24—H24	119.5
C4—C10—C9	116.01 (16)	O1—C25—H25A	109.5
C9—C10—C5	115.74 (16)	O1—C25—H25B	109.5
O3—C11—C1	109.27 (11)	O1—C25—H25C	109.5
O3—C11—C12	110.59 (11)	H25A—C25—H25B	109.5
O3—C11—C18	101.39 (10)	H25A—C25—H25C	109.5
C1—C11—C18	102.50 (10)	H25B—C25—H25C	109.5
C12—C11—C1	118.16 (11)	O2—C26—H26A	109.5
C12—C11—C18	113.41 (11)	O2—C26—H26B	109.5
C13—C12—C11	121.58 (13)	O2—C26—H26C	109.5
C17—C12—C11	119.66 (12)	H26A—C26—H26B	109.5
C17—C12—C13	118.69 (14)	H26A—C26—H26C	109.5
C12—C13—H13	119.6	H26B—C26—H26C	109.5
C14—C13—C12	120.74 (15)	O3—C27—H27A	109.5
C14—C13—H13	119.6	O3—C27—H27B	109.5
C13—C14—H14	120.8	O3—C27—H27C	109.5
C15—C14—C13	118.43 (16)	H27A—C27—H27B	109.5
C15—C14—H14	120.8	H27A—C27—H27C	109.5
F1—C15—C14	118.61 (17)	H27B—C27—H27C	109.5
			
F1—C15—C16—C17	−176.86 (14)	C9—C1—C11—O3	87.60 (13)
F2—C22—C23—C24	179.41 (14)	C9—C1—C11—C12	−144.84 (12)
O1—C2—C3—C4	174.48 (16)	C9—C1—C11—C18	−19.37 (14)
O2—C7—C8—C9	−177.25 (13)	C9—C8—C18—O4	−137.95 (12)
O2—C7—C8—C18	−1.9 (2)	C9—C8—C18—C11	−19.14 (13)
O3—C11—C12—C13	148.52 (13)	C9—C8—C18—C19	100.53 (13)
O3—C11—C12—C17	−34.66 (17)	C10—C5—C6—C7	2.6 (3)
O3—C11—C18—O4	31.44 (13)	C11—C1—C2—O1	−5.4 (3)
O3—C11—C18—C8	−90.22 (11)	C11—C1—C2—C3	172.69 (15)
O3—C11—C18—C19	149.93 (10)	C11—C1—C9—C8	8.32 (16)
O4—C18—C19—C20	34.99 (16)	C11—C1—C9—C10	−171.21 (13)
O4—C18—C19—C24	−146.74 (13)	C11—C12—C13—C14	178.65 (13)
C1—C2—C3—C4	−3.5 (3)	C11—C12—C17—C16	−177.26 (13)
C1—C9—C10—C4	−3.6 (2)	C11—C18—C19—C20	−85.53 (14)
C1—C9—C10—C5	175.15 (14)	C11—C18—C19—C24	92.74 (15)
C1—C11—C12—C13	21.59 (19)	C12—C11—C18—O4	−87.12 (13)
C1—C11—C12—C17	−161.59 (13)	C12—C11—C18—C8	151.21 (11)
C1—C11—C18—O4	144.36 (11)	C12—C11—C18—C19	31.37 (15)
C1—C11—C18—C8	22.70 (12)	C12—C13—C14—C15	−1.3 (2)
C1—C11—C18—C19	−97.14 (12)	C13—C12—C17—C16	−0.3 (2)
C2—C1—C9—C8	−178.37 (13)	C13—C14—C15—F1	178.26 (14)
C2—C1—C9—C10	2.1 (2)	C13—C14—C15—C16	−0.8 (3)
C2—C1—C11—O3	−84.26 (19)	C14—C15—C16—C17	2.2 (3)
C2—C1—C11—C12	43.3 (2)	C15—C16—C17—C12	−1.6 (2)
C2—C1—C11—C18	168.77 (16)	C17—C12—C13—C14	1.8 (2)
C2—C3—C4—C10	1.9 (3)	C18—C8—C9—C1	7.86 (16)
C3—C4—C10—C5	−177.04 (17)	C18—C8—C9—C10	−172.60 (13)
C3—C4—C10—C9	1.6 (2)	C18—C11—C12—C13	−98.36 (15)
C5—C6—C7—O2	174.01 (15)	C18—C11—C12—C17	78.45 (15)
C5—C6—C7—C8	−3.3 (2)	C18—C19—C20—C21	177.64 (13)
C6—C5—C10—C4	179.75 (16)	C18—C19—C24—C23	−177.47 (13)
C6—C5—C10—C9	1.2 (2)	C19—C20—C21—C22	−0.2 (2)
C6—C7—C8—C9	0.3 (2)	C20—C19—C24—C23	0.8 (2)
C6—C7—C8—C18	175.57 (14)	C20—C21—C22—F2	−179.28 (13)
C7—C8—C9—C1	−175.85 (13)	C20—C21—C22—C23	1.0 (2)
C7—C8—C9—C10	3.7 (2)	C21—C22—C23—C24	−0.8 (3)
C7—C8—C18—O4	46.4 (2)	C22—C23—C24—C19	−0.1 (2)
C7—C8—C18—C11	165.18 (15)	C24—C19—C20—C21	−0.7 (2)
C7—C8—C18—C19	−75.15 (19)	C25—O1—C2—C1	−170.50 (16)
C8—C9—C10—C4	176.89 (14)	C25—O1—C2—C3	11.5 (3)
C8—C9—C10—C5	−4.3 (2)	C26—O2—C7—C6	17.1 (2)
C8—C18—C19—C20	160.74 (12)	C26—O2—C7—C8	−165.49 (14)
C8—C18—C19—C24	−20.99 (18)	C27—O3—C11—C1	66.72 (15)
C9—C1—C2—O1	−176.62 (13)	C27—O3—C11—C12	−64.99 (15)
C9—C1—C2—C3	1.5 (2)	C27—O3—C11—C18	174.44 (11)

### Hydrogen-bond geometry (Å, º)

*Cg*2, *Cg*5, *Cg*8 and *Cg*9 are the centroids of the rings (C1–C4,C9,C10), (C19–C24), (C1–C10) and (C1–C8,C10,C11,C18), respectively.

**Table:**

*D*—H···*A*	*D*—H	H···*A*	*D*···*A*	*D*—H···*A*
O4—H4···O3	0.83 (3)	2.00 (3)	2.5119 (18)	119 (3)
C24—H24···*Cg*9	0.95	2.90	3.4960 (17)	122
C20—H20···O4^i^	0.95	2.49	3.3554 (17)	151
C4—H4*A*···F1^ii^	0.95	2.44	3.189 (3)	135
C26—H26*C*···*Cg*2^iii^	0.95	2.70	3.614 (2)	155
C26—H26*C*···*Cg*8^iii^	0.95	2.85	3.747 (2)	153
C26—H26*C*···*Cg*9^iii^	0.95	2.91	3.848 (2)	160
C25—H25*B*···*Cg*5^iv^	0.95	2.81	3.527 (3)	131

Symmetry codes: (i) −*x*, −*y*+1, −*z*+1; (ii) *x*, *y*, *z*+1; (iii) −*x*, −*y*+1, −*z*+2; (iv) *x*+1, *y*, *z*.
