# Supplementary material for: Crystal structure of 1,2-bis­(4-fluoro­phen­yl)-1-hy­droxy-2,3,8-tri­meth­oxy­acenaphthene: formation of a five-membered intra­molecular O—H⋯O hydrogen-bonded ring
Source: Acta Crystallogr E Crystallogr Commun. 2021 Jan 26;77(Pt 2):175–9. doi: 10.1107/S2056989021000669 (PMC7869547; doi:10.1107/S2056989021000669)
Supplement: Supplementary file 3 [file e-77-00175-sup3.pdf]

ピーク検出 - 20201204-IRきれい.jws

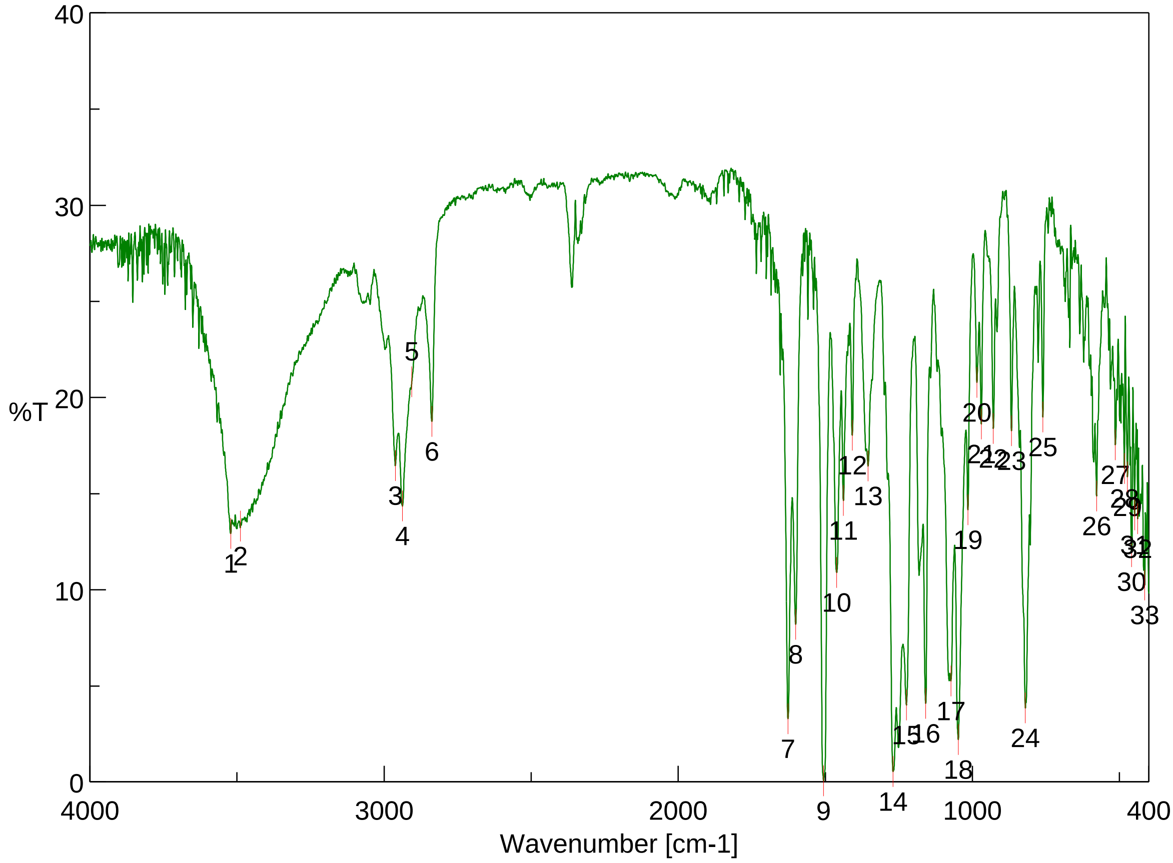

[コメント情報]

試料名	
コメント	
測定者	
所属	
会社	東京農工大学

[データ情報]

作成日時	2021/01/05 15:13
データタイプ	等間隔データ
横軸	Wavenumber [cm-1]
縦軸	%T
スタート	399.675 cm-1
エンド	4000.12 cm-1
データ間隔	0.482117 cm-1
データ数	7469

[測定情報]

機種名	FT/IR-4100typeA
シリアル番号	B041461016
測定日時	2021/01/05 15:04
光源	標準光源
検出器	TGS
積算回数	16
分解	2 cm-1
ゼロフィリング	On
アポダイゼーション	Cosine
ゲイン	Auto (16)
アパーチャー	Auto (5 mm)
スキャンスピード	Auto (2 mm/sec)
フィルタ	Auto (30000 Hz)

[ピーク検出結果]

No.	位置	強度	No.	位置	強度
1	3521.38	12.9168	2	3488.11	13.3012
3	2961.64	16.4395	4	2937.54	14.3567
5	2905.72	20.8022	6	2837.74	18.7419
7	1627.14	3.28151	8	1600.63	8.18177
9	1505.65	0.051195	10	1461.3	10.8854
11	1438.15	14.6274	12	1408.26	18.0201
13	1354.75	16.4272	14	1269.41	0.52771

[ピーク検出結果]

No.	位置	強度	No.	位置	強度
15	1224.09	3.99425	16	1158.53	4.07744
17	1073.19	5.24816	18	1048.12	2.19135
19	1015.34	14.144	20	984.964	20.7796
21	969.537	18.6127	22	928.557	18.372
23	867.328	18.2541	24	820.081	3.83788
25	760.298	18.98	26	577.576	14.8714
27	513.936	17.5286	28	483.081	16.2951
29	472.474	15.8654	30	458.975	11.9758
31	448.369	13.8755	32	437.762	13.6871
33	414.138	10.2385			
