# Supplementary material for: Crystal structure of 1,2-bis­(4-fluoro­phen­yl)-1-hy­droxy-2,3,8-tri­meth­oxy­acenaphthene: formation of a five-membered intra­molecular O—H⋯O hydrogen-bonded ring
Source: Acta Crystallogr E Crystallogr Commun. 2021 Jan 26;77(Pt 2):175–9. doi: 10.1107/S2056989021000669 (PMC7869547; doi:10.1107/S2056989021000669)
Supplement: Supplementary file 5 [file e-77-00175-sup5.pdf]

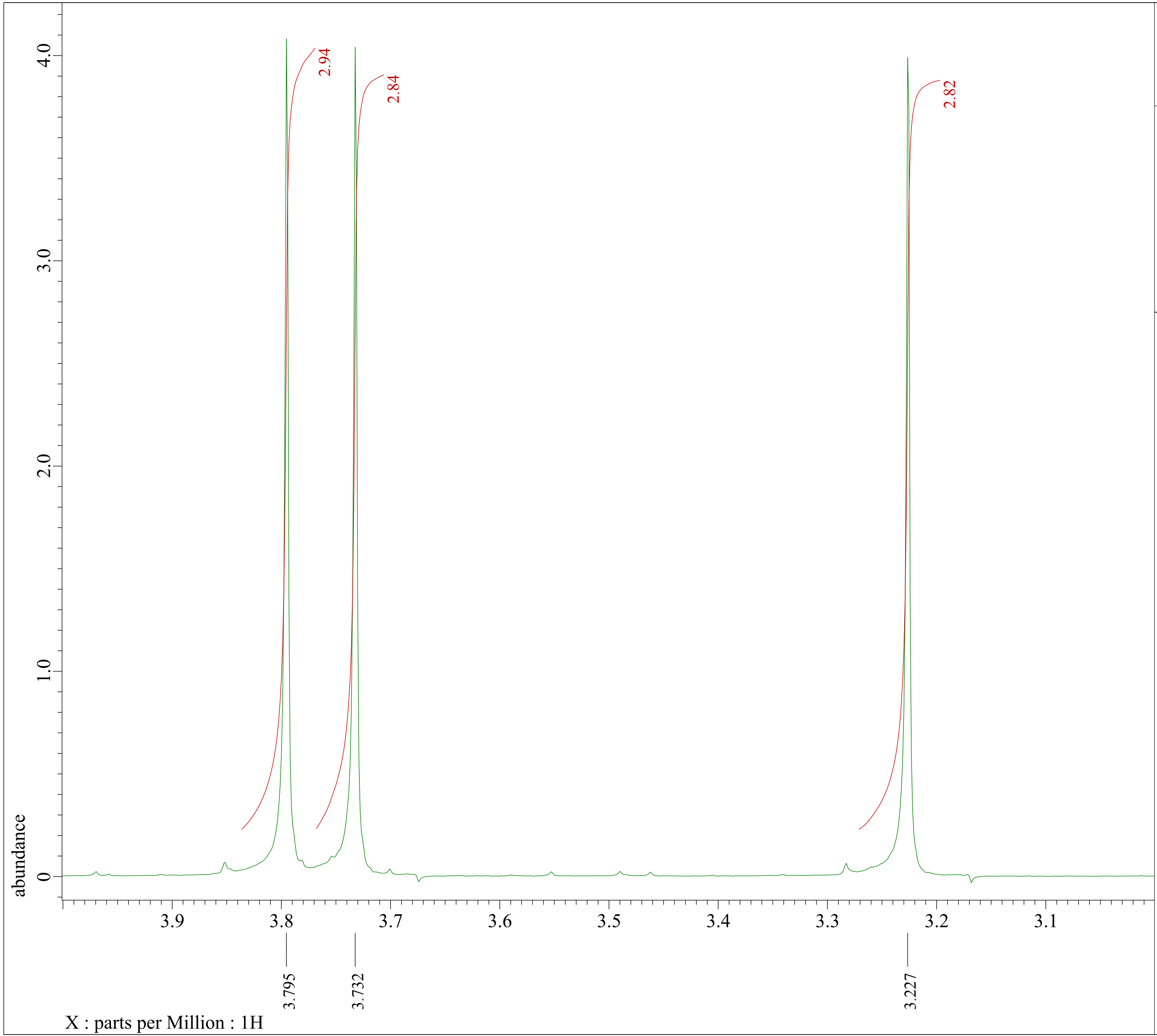

JEOL

```
---- PROCESSING PARAMETERS ----
dc_balance( 0, FALSE )
sexp( 0.2[Hz], 0.0[s] )
trapezoid3( 0[%], 80[%], 100[%] )
zerofill( 1 )
fft( 1, TRUE, TRUE )
machinephase
ppm
```

```
Filename           = 20201204-PTLC-II-3.jdf
Author             = delta
Experiment         = single_pulse.ex2
Sample_Id          = S#561308
Solvent            = CHLOROFORM-D
Actual_Start_Time  = 24-DEC-2020 20:13:57
Revision_Time      =  6-JAN-2021 14:07:44

Comment            = single_pulse
Data_Format        = 1D COMPLEX
Dim_Size           = 13107
X_Domain           = 1H
Dim_Title          = 1H
Dim_Units          = [ppm]
Dimensions         = X
Site               = ECX 300
Spectrometer       = JNM-ECX300

Field_Strength     = 7.0586013[T] (300[MHz])
X_Acq_Duration     = 2.90717696[s]
X_Domain           = 1H
X_Freq             = 300.52965592[MHz]
X_Offset           = 5[ppm]
X_Points           = 16384
X_Prescans         = 1
X_Resolution       = 0.34397631[Hz]
X_Sweep            = 5.63570784[kHz]
Irr_Domain         = 1H
Irr_Freq           = 300.52965592[MHz]
Irr_Offset         = 5[ppm]
Tri_Domain         = 1H
Tri_Freq           = 300.52965592[MHz]
Tri_Offset         = 5[ppm]
Clipped            = FALSE
Scans              = 8
Total_Scans        = 8

Relaxation_Delay   = 5[s]
Recvr_Gain         = 36
Temp_Get           = 460.0[dC]
X_90_Width         = 11.8[us]
X_Acq_Time         = 2.90717696[s]
X_Angle            = 45[deg]
X_Atn              = 1.8[dB]
X_Pulse            = 5.9[us]
Irr_Mode           = Off
Tri_Mode           = Off
Dante_Presat       = FALSE
Initial_Wait       = 1[s]
Repetition_Time    = 7.90717696[s]
```
